# Current Progress in Clinical Research in Secondary Prevention and Early Detection of Colorectal Cancer

**DOI:** 10.3390/cancers17030367

**Published:** 2025-01-23

**Authors:** Olga Partyka, Monika Pajewska, Aleksandra Czerw, Andrzej Deptała, Dominika Mękal, Katarzyna Sygit, Dariusz Kowalczyk, Elżbieta Cipora, Mateusz Kaczmarski, Lucyna Gazdowicz, Grażyna Dykowska, Zofia Sienkiewicz, Tomasz Banaś, Krzysztof Małecki, Elżbieta Grochans, Szymon Grochans, Anna Maria Cybulska, Daria Schneider-Matyka, Ewa Bandurska, Tomasz Bandurski, Jarosław Drobnik, Piotr Pobrotyn, Michal Marczak, Remigiusz Kozlowski

**Affiliations:** 1 Department of Health Economics and Medical Law, Medical University of Warsaw, 02-091 Warsaw, Poland; 2 Department of Economic and System Analyses, National Institute of Public Health NIH—National Research Institute, 00-791 Warsaw, Poland; 3 Department of Oncology Propaedeutics, Medical University of Warsaw, 01-445 Warsaw, Poland; 4 Faculty of Health Sciences, Calisia University, 62-800 Kalisz, Poland; 5 Medical Institute, Jan Grodek State University in Sanok, 38-500 Sanok, Poland; 6 Department of Nursing, Social and Medical Development, Medical University of Warsaw, 02-091 Warsaw, Poland; 7 Department of Radiotherapy, Maria Sklodowska-Curie Institute-Oncology Centre, 31-115 Cracow, Poland; 8 Department of Radiotherapy for Children and Adults, University Children’s Hospital of Cracow, 30-663 Cracow, Poland; 9 Department of Nursing, Pomeranian Medical University in Szczecin, 71-210 Szczecin, Poland; 10 Department of Clinical Nursing, Faculty of Health Sciences, Pomeranian Medical University in Szczecin, 71-210 Szczecin, Poland; 11 Center for Competence Development, Integrated Care and e-Health, Medical University of Gdansk, 80-204 Gdansk, Poland; 12 Division of Radiology Informatics and Statistics, Medical University of Gdansk, 80-210 Gdansk, Poland; 13 Department of Family Medicine, Faculty of Medicine, Wroclaw Medical University, 51-141 Wroclaw, Poland; 14 Remedial Specialistic Clinic, “Pulsantis Sp. z o.o”, 53-238 Wroclaw, Poland; 15 Department of Innovation, Merito University in Poznan, 61-895 Poznan, Poland; 16 Department of Management and Logistics in Healthcare, Medical University of Lodz, 90-131 Lodz, Poland

**Keywords:** screening, secondary prevention, colorectal cancer, CRC

## Abstract

Colorectal cancer (CRC) is the second leading cause of cancer-related deaths globally. The risk of disease increases with age, as most CRC patients are over 50 years old. Due to the progressive aging of societies in high-income countries, the problem of CRC will increase. This makes the development of new early detection methods and the implementation of effective screening programs crucial. Key areas of focus include raising population awareness about the importance of screening, educating high-risk populations, and improving and developing early diagnostic methods. The primary goal of this review is to provide a concise overview of recent trends and progress in CRC secondary prevention based on available information from clinical trials.

## Simple Summary

Colorectal cancer is one of the leading causes of cancer-related death worldwide. Prevention remains the most effective strategy to reduce mortality and mitigate its impact, but public participation in screening programs varies significantly. Advances in science and technology have provided new opportunities for action, but navigating the vast amount of information on early detection can be overwhelming for those interested in the topic. This review presents an overview of the current trends in clinical research related to secondary prevention of CRC.

## 1. Introduction

### 1.1. Epidemiology and Risk Factors

The World Health Organization reports that colorectal cancer (CRC) is the third most common cancer and the second leading cause of death among oncological diseases in the world [1]. It is important to know that the majority of colorectal cancers are sporadic when looking at epidemiological data, but the incidence rate is increasing. Because of this, it is important to take action in the field of early detection and screening.

Risk factors for the disease can be divided into two main categories: modifiable (such as lifestyle) and non-modifiable (such as genetic, family history, and age) risk factors [2]. The risk of developing colorectal cancer increases with age, and it most often occurs in people over 50 years of age. According to research, sex may play a role in the diagnosis of colorectal cancer. In a study by Regula et al., the male sex was identified as a predictive factor for detecting advanced cancer in screening. The remaining variables were an age over 49 and a family history of colorectal cancer [3].

There is an increased risk of developing colorectal cancer in people with a positive familial oncological history. The risk of CRC is much higher in people with a first-degree relative diagnosed with the disease before the age of 60 [4].

Modifiable risk factors related to one’s lifestyle primarily include diet and obesity. Eating a diet rich in saturated fat significantly increases the risk of developing CRC. Among other modifiable risk factors, smoking tobacco products increases the risk of developing the disease by 2–3 fold, and alcohol consumption also play an important role; daily consumption of 2–3 servings of alcohol increases the risk of CRC by 20% [5,6,7].

### 1.2. Screening Methods and Participation Rate

The American Cancer Society recommends screenings for people over the age of 45 and up to the age of 75. The decision to screen people aged 76–85 should be based on current health and past medical history, and people over the age of 85 should no longer be screened [8]. Adverse events related to colorectal cancer screening are few and result mainly from the use of minimally invasive colonoscopy [8]. One of the basic methods used in the early detection of colorectal cancer is colonoscopy. This method is relatively safe, but it is an invasive method. The incidence of perforation is less than 1/1000 cases, and in most cases, it is caused by polypectomy and results directly from the procedure [9].

In Europe, according to the recommendations of the European Commission, colorectal cancer screenings should cover people between 49 and 69 years of age. The basic recommended test is a quantitative stool immunochemical test (FIT) performed before the patient undergoes a colonoscopy or endoscopy. The FIT is a noninvasive method which can be performed at the patient’s home, which increases comfort and accessibility to tests and reduces the costs of maintaining the system [10].

The age range of those recommended for screening has decreased from previous recommendations. The majority of CRC cases are in the elderly, but there is an apparent increase in early-onset colorectal cancer (EO-CRC) in people under 50. This is particularly evident in high-income countries [11]. In the US, the incidence of CRC in people younger than 50 years has increased by 1.4% annually, and it has also been documented in most European countries; in the time period of 2004–2016, the annual percent changes in EO-CRC incidence were 7.9% in those aged 20–29, 4.9% in those aged 30–39, and 1.6% in those aged 40–49 [12]. In total, 30% of all cases can be attributed to hereditary cancer syndromes and various hamartomatous polyposis conditions [13]. The mechanism of this phenomenon is not known. Some researchers believe that it is caused by a diet high in fat, obesity, and low physical activity, but the exact mechanism is not known. According to the results obtained by Bretthauer et al. in a randomized study conducted in Poland, Sweden, and Norway with a sample of 84,585 people, the risk of CRC was lower in people who took part in colonoscopy screenings compared with people who did not receive invitation to colonoscopies. The risk of developing CRC in the study group after a 10 year follow-up was 0.98% versus 1.20% in the control group [14]. Screening is a cost-effective tool for reducing the burden of colorectal cancer. Most OECD countries have screening programs for select groups of cancers which can be effectively detected and treated at an early stage for targeted high-risk populations. An example of existing solutions for early detection of colorectal cancer is the Early Detection of Colorectal Cancer program conducted in Poland since 2000 as part of the National Cancer Prevention Program (NPZChN), with the current edition covering the years 2016–2024. The program assumed that colonoscopies would be performed on an opportunistic and population basis (by invitation every 10 years), but due to the availability of centers performing colonoscopy screenings and concerns about the examination itself, the assumptions of the program required changes (during the COVID-19 pandemic, only 10% of the eligible population took part). Since 2024, the FIT has been introduced into the screening program. The advantage of the test, apart from its high sensitivity and specificity, is its noninvasiveness and the lack of a need for prior preparation for the test. An invitation to an FIT combined with a reminder was addressed to people aged 50–69. If the patient ignored the invitation or obtained a negative result, then the patient would receive another invitation in 2 years. If the FIT was positive, then a colonoscopy was performed. As part of the program, colonoscopy screening was addressed to people aged 40–65 with a history of familial CRC, with the history taking itself becoming the responsibility of a GP or an occupational medicine physician. The Polish program also includes screening of people aged 25–49 from families with a genetic risk of hereditary CRC (in the case of HNPCC, colonoscopies should be repeated every 2–3 years, and in people with FAP, colonoscopies should be performed every year) [15].

According to OECD data, the population-based CRC screening program in Europe covers from 13.3% to 73% of the population of a given country [16]. Such a large difference indicates the need for further measures to improve participation and therefore early detection of colorectal cancer. Despite existing well-developed screening tools, only 4 out of 10 CRC cases are detected at an early stage [17]. According to studies, there are many reasons why people do not adhere to screenings. In a study by Gordon and Green, the main reasons for not following a doctor’s recommendations and participating in the FIT were discomfort, disgust, or embarrassment (59.6%); thinking it unnecessary (32.9%); fatalism or fear; and thinking they are too difficult to follow [18]. Richardson et al. provided examples such as low motivation and understanding of the screening procedure itself [19]. The fear of being diagnosed with cancer also plays a role [20]. For this reason, it is important to improve screening methods, mainly to identify people at high risk (e.g., individuals with a history of colorectal polyps, inflammatory bowel disease, familial adenomatous polyposis, or first-degree relatives) and to include them in screening programs.

The increasingly rapid progress of science, work on new diagnostic methods, and a large number of ongoing studies are positive phenomena for people involved in public health, but the amount of available information may be difficult to assimilate. For this reason, we decided to collect in one place the most important information from the point of view of public health, which concisely presents current trends in screening for colorectal cancer, which is one of the dominant cancers in the world.

The aim of this review is to present current trends in research in the area of screening for and early detection of colorectal cancer to provide information for researchers interested in secondary cancer prevention. The knowledge collected in our work can serve as a valuable source of information for people interested in the topic of secondary prevention of colorectal cancer.

## 2. Materials and Methods

The basis for the review was information collected from the world’s largest clinical trial registry (ClinicalTrial.Gov).

Three main categories were adopted and analyzed:

(a) Population interventions aimed at increasing participation in screenings: All trials aimed at increasing participation in screenings were included in this category;
(b) Educational interventions: We included trials and interventions aimed at increasing knowledge about risk factors, countermeasures, available diagnostic methods, health literacy among patients, and increasing knowledge about CRC, the functioning of existing solutions in an organization, and the operation of a health care system from the perspective of the healthcare provider,
(c) Development, improvement, and application of early detection methods: This study included trials on the development of new diagnostic methods and procedures, improvement of existing methods and procedures, and improving the quality of methods and patient comfort.

The analysis included interventional studies, the main goal of which was early detection of colorectal cancer and in which the study group consisted of healthy people without symptoms. Studies relating to other cancers and studies targeting already-developed cancer were excluded from the analysis. In order to present the current trend in research, the search period was narrowed to 2019–2023. This analysis included studies which had a complete and active status by 31 December 2023.

**Figure 1:**
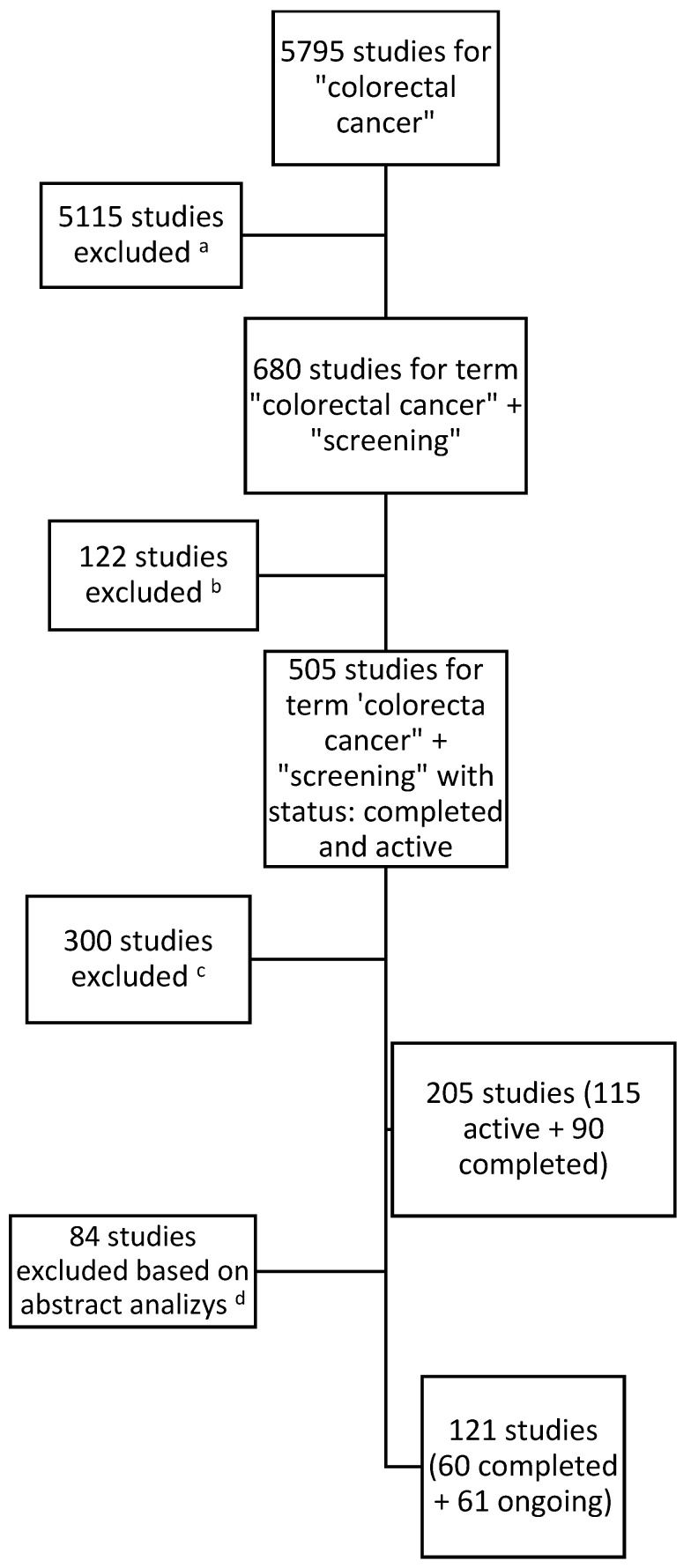
Results for searching and including studies in the analysis. ^a^ Studies with no connection with the keywords ‘screening’ or ‘early detection’. ^b^ Studies with the status terminated, suspended, withdrawn, or unknown in the database on the last day of the analyzed time period. ^c^ Studies which did not fit in the period of time from 1 January 2019 to 31 December 2023. ^d^ Studies which did not cover the topic of population screening (e.g., focused on patients with diagnosed CRC or methods of treatment and not methods of early detection or screening methods) and studies which, after reading the study information, did not fit into any category.

Below (Figure 1) is a process tree for including studies in the analysis:

The first data search identified a total of 205 studies, of which 115 were active and 90 studies had been completed. As a result of the analysis of the study descriptions by two researchers, those studies which did not fall within the scope of the analysis after analyzing the texts were excluded. (The studies which did not fit into any category, did not consist of healthy participants, or focused on patients with cancer were excluded.) The researchers then compared the results and, by consensus, developed a list of studies for inclusion. Ultimately, 121 studies were included in the analysis.

Table 1 presents the results of the analysis of the collected material regarding research on early detection of colorectal cancer in the studied period.

In the analyzed period, the number of studies aimed at colorectal cancer screenings has been systematically increasing, although in 2023, there was a slight decrease, perhaps due to the completion of previous studies. This indicates that researchers are actively interested in the growing problem of early detection of colorectal cancer. In the period from 1 January 2019 to 31 December 2023, 121 clinical trials were registered in the database (displayed in the database as of 15 June 2024). This number may vary slightly during other viewings. This is due to the editing of research statuses by their creators. However, this does not affect the overall picture of CRC screening research. The purpose of these trials fell into at least one of the three research categories assumed for the analysis. During the analyzed period, 60 studies were started and completed, obtaining the “completed” status, while 61 studies had the “ongoing” status as of the last day of the analyzed period (31 December 2023).

## 3. Colorectal Cancer Secondary Prevention

### 3.1. Population Interventions Aimed at Increasing Participation in Screenings

Research shows that in the case of colorectal cancer, a postal invitation combined with another form of reminder, mainly by telephone, translates into a higher participation rate compared with a postal invitation alone, ranging between 1 and 1.76 times greater. The results of some studies indicate that the inclusion of a face-to-face intervention, compared with an invitation alone, increases patient participation in screenings (RR = 1.29, 95% CI: 1.14–1.45). Two studies compared the use of prior notifications about the possibility of participating in CRC screenings (automatic text message notifications and a telephone call from a treatment facility) versus standard invitations (e.g., letter). Both studies showed similar results, indicating a 14% increase in adherence to screening recommendations in the case of the method with prior notification (RR = 1.14, 95% CI: 1.08–1.19) [23,34]. The clinical trials included in the analysis focused on adapting or modifying existing tools such as postal and telephone invitations and information materials with a proactive attitude (e.g., attaching a self-administered FIT kit (stool immunochemical test) to the invitation to the study) [22,24,42].

Studies fitting into the first category often occurred in combination with the other two groups, most often with the second category. An example of activities in this category is the study ‘Audio and Video Brochures for Increasing Colorectal Cancer Screening Among Adults Living in Appalachia’ (NCT05810714), which was aimed at a geographically excluded community. The basis of the study was information brochures in the form of audiovisual recordings aimed at increasing the percentage of people from the region taking part in colorectal cancer screening using an FIT kit. The audiovisual recordings were intended to be easier to understand for people with a limited level of health literacy, which in the end would translate into a higher return of FIT kits for testing [25]. The results of study NCT05810714 show that the return of FIT kits was higher among patients who were sent the kits in combination with a video brochure than in the group of patients who did not receive additional materials ((OR: 3.1; 95% CI: 1.02, 9.2; *p* = 0.046). However, the overall rate of recovery still requires further improvement, especially among a health-disadvantaged community [27]. Another ongoing study in 2021–2022 covering CRC and cervical cancer was ‘Multilevel Intervention Based on Colorectal Cancer (CRC) and Cervical Cancer Self-screening in Rural, Segregated Areas’ (NCT04471194), which was also aimed at a geographically excluded group: inhabitants of rural areas and areas inhabited by ethnic people. The study also included increasing the percentage of return of FIT kits [43]. An example of a study aimed at overall improvement in CRC screening uptake was the study ‘Health Service Intervention for the Improvement of Access and Adherence to Colorectal Cancer Screening’ (NCT04607291) [36]. The study used the Witness CARES Services protocol, which is used to optimize clinical services. Patients who are not prepared for a colonoscopy or stool testing receive educational materials, namely messages and videos with information about colon screening tests, electronically or by post and then make an appointment by phone within 2 weeks. Patients who want to undergo a colonoscopy examination receive assistance with the path (e.g., setting an appointment, preparing materials and the process, and transport). Patients who want to take part in a stool test are assisted by a coordinator who facilitates the test. Another study, ‘Optimizing Colorectal Cancer Screening Participation’ (NCT04292366), aimed to demonstrate the effectiveness of a three- and four-step invitation protocol compared with two-step procedures by combining pre-notifications and reminders. The iinitial notifications were sent to one of the groups 10 days before the intervention. Additionally, they received an invitation and one additional reminder. In the case of the second group, the notification interval was different, having an invitation and two reminders: one after 45 days and the other 3 months after the invitation. The third group received an initial notification, an invitation, a reminder 45 days later, and a reminder 3 months after the invitation. The control group received a regular invitation and one reminder 45 days after the invitation. In all cases, the reminders were delivered digitally [35]. The results from the study showed an increase in the overall participation rate of 2.9% (95% CI: 1.8–4.0), rising from 66.9% to 69.8%, and in the group with additional reminder by 2.7% (95% CI: 1.1; 4.2) among men and by 1.1% (95% CI: −0.3; 2.5) among women [44]. People from groups at risk of social inequalities in health resulting from ethnicity, race, or place of residence, which limit access to screenings, are included in the research area particularly often [38,41]. According to studies, the participation rate in CRC screenings was lower in the patients among the youngest age group, people with lower levels of education, and immigrants. Also, living in a rural area was associated with lower use of colonoscopies (ORs from 0.76 (95% CI: 0.56, 0.96) to 0.87 (95% CI: 0.81, 0.93)). Because of this, the communication tools used to improve adherence to screenings should be tailored to the participant group the system is trying to reach [30].

Most studies targeted the population. However, a different approach was demonstrated in the study ‘General Practitioners and Participation Rate in ColoRectal Cancer Screening’ (AMDepCCR) (NCT04492215). It focused on primary care physicians, on whom the CRC screening program in France is based. The aim of the study was to create a training program for GPs based on motivational interviewing (MI) techniques for the promotion of colorectal cancer screenings. Motivational interviewing is a directive, patient-centered counseling style for eliciting behavior changes by helping clients explore and resolve ambivalence [37].

### 3.2. Educational Interventions

The second category included studies aimed at increasing knowledge and education in the field of early detection of colorectal cancer. Health literacy was an issue of particular interest to researchers. The main goal of the activities was to improve the understanding and use of health information in the decision-making process and compliance with medical recommendations. Educational activities were also aimed at reducing patients’ fears and reluctance to undergo screenings, especially colonoscopies, and preparing appropriately for the tests. Research in the second category, including educational interventions, was mainly carried out in two groups: educational activities among patients, particularly those at high risk of developing colorectal cancer, and activities aimed at increasing the knowledge of healthcare providers about early detection and CRC risk factors, especially among primary care physicians [21,31,45,46].

These studies targeted select communities, such as ‘Screen to Save 2: Rural Cancer Screening Educational Intervention’ (NCT04414306), which was aimed at a select group of patients. The basis of the educational program was the Screen to Save 2 protocol implemented in two formats: a traditional form of contact via stationary contact and an online format. Education in both formats was based on a set of key messages including information about colorectal cancer, risk factors, prevention, screening recommendations, and screening options. Key messages from the National Cancer Institute have been adapted to the local NH and VT contexts, including information about local options for colorectal cancer screening [39]. Another study aimed at health education of a selected local community is the study ‘The PRIME-CRC Trial to Promote CRC Screening in Rural Communities’ (NCT04313114), which apart from increasing the level of participation in research also assumes health education and improving the health literacy of local communities with difficult access to health care [24]. The systematic review by Zhang et al. indicates insufficient health education as one of the barriers hindering the use of screenings [29]. Another goal of health education in CRC is to reduce fear of screenings, especially colonoscopies. An example of such a study is NCT05458986, which aimed to use video intervention to alleviate fear of colonoscopies. The results of other studies, such as Shahrbabaki et al., showed that education before a test reduces fear, as in the study group, the values were lower after educational intervention than before it (t = −6.01; *p* < 0.001) [47].

Some of the studies were aimed at selected ethnic groups, such as the ‘Educate, Assess Risk and Overcoming Barriers to Colorectal Screening Among African Americans’ study (NCT03640208), which was aimed at groups affected by health inequalities. African Americans, according to the data, have a higher mortality rate than all other social groups in the USA. The study involved the use of an educational session supported by visual aids, covering such issues as risk factors, what colorectal cancer is, prevention, and screening instructions [41].

The risk of developing CRC increases in people closely related to the patient. The aim of the ongoing study ‘Comparing the Effectiveness of Written vs. Verbal Advice for First Degree Relatives of Colorectal Cancer Patients’ (NCT06242197) is to evaluate the effectiveness of educating first-degree relatives about the risk of colorectal cancer in written and verbal form [48]. The study ‘Impact of Colorectal Cancer and Nutrition Education Program Among Minority Patients with Type 2 Diabetes’ (NCT05765214) aimed to develop an educational program aimed at increasing knowledge about CRC prevention among patients with type 2 diabetes. The authors wanted to answer questions about the factors related to CRC screening among diabetics and check the impact of patient-oriented health and nutrition education, tailored to the cultural factors in which they live, on the risk of developing CRC [45].

An example of a study aimed at reducing fear of colonoscopic screenings was the study ‘A Video Intervention to Decrease Patient Fear of Colonoscopy After a Positive Fecal Immunochemical Test’ (NCT05458986). The aim of the study was to compare an educational intervention using a video recording and an intervention without visual support. The starting point for the study was the low incidence of colonoscopy and positive FIT results. The preparation for the colonoscopy examination itself was also the subject of research. Appropriate patient preparation not only affects the course of the examination itself but also reduces concerns about the procedure and increases satisfaction [49]. An example of such a study is ‘An Interactive Video Educational Tool Improves the Quality of Bowel Preparation for Colonoscopy’ (NCT04491565), where one group received instructions in the form of verbal and written instructions and one group additionally had access to an interactive online recording [21].

### 3.3. Development and Application of Early Detection Methods

The registered clinical trials focused on two factors. (1) Some focused on blood tests based on the detection of circulating cell-free DNA (cfDNA), including the circulating tumor DNA (ctDNA) of colorectal cancer. According to CMS guidelines, a blood test for CRC, in order to be approved for use, must achieve the thresholds of 90% specificity and 74% sensitivity compared with the accepted standard, which is colonoscopy [40]. According to studies, ctDNA detection using liquid biopsy could be less invasive and safer than tissue biopsy [28,40]. The advantage of liquid biopsy is the short examination time (7–10 days compared with 2–3 weeks needed for classic histopathological examinations). In a case–control study by Brenne et al., based on 106 samples taken from people diagnosed with CRC and 106 control samples in which methylated ctDNA markers were detected, it was found that it is possible to detect ctDNA markers up to 2 years before clinical diagnosis in a population resembling screening conditions. This indicates the possibility of using ctDNA in early CRC detection programs [32]. An example of a study on a new blood cancer detection test which was active in the analyzed period is the prospective, multicenter PREEMPT CRC study (NCT04369053), which is using AI to create a platform that will integrate the signals (peripheral blood material) of tumor DNA, DNA of tumor microenvironment cells, and DNA of immune response cells (multiomics), which would improve detection sensitivity [50]. The starting point of the ‘Identification of New Diagnostic Protein Markers for Colorectal Cancer’ study (EXOSCOL01) (NCT04394572) was the release of extracellular vesicles by cancer cells containing proteins, mRNA, and DNA. These extracellular vesicles are of two types: exosomes (40–100 nm in diameter) formed by the budding of endosomal membranes and microvesicles (100–1000 nm in diameter) formed by budding of the cell membrane. Exosomes contain transmembrane proteins on their surface, called tetraspanins (CD9, CD63, and CD81). The research hypothesis assumed the use of these proteins as diagnostic markers in the detection of CRC [51]. The ‘A Multicenter Clinical Trial of Stool-based DNA Testing for Early Detection of Colon Cancer in China’ study (NCT04722055) focused on the development of a new screening kit which could replace FOBTs: the Human Multigene Methylation Detection Kit (fluorescent PCR). The tool is designed to qualitatively detect the methylation levels of multiple genes in human stool samples in vitro using quantitative methylation-specific PCR (qMSP) [26]. The results of another study, ‘Clinical Validation of An Optimized Multi-Target Stool DNA (Mt-sDNA 2.0) Test, for Colorectal Cancer Screening “BLUE-C”’ (NCT04144738), focused on clinical validation of optimized multi-target stool DNA. The sensitivity for colorectal cancer was 93.9% (95% CI: 87.1–97.7), and the specificity for advanced neoplasia was 90.6% (95% CI: 90.1–91.0). The sensitivities appeared to be higher with the next-generation test than with the FIT for the detection of colorectal cancers from stages I to III (92.7% versus 64.6%), proximal colorectal cancers (88.2% versus 58.8%), and distal colorectal cancers (96.9% versus 71.9%). Studies have indicated an aggregate sensitivity for the test of 65% (95% CI: 57–71%) [33,52,53]. A number of factors can contribute to the sensitivity and specificity of a genetic screening test. These include the DNA quality, molecular technology, and specific genetic changes chosen for analysis. The stool DNA test is based on the collection of a single stool sample, and there are no dietary restrictions, which affects the patient’s comfort. The DNA itself is derived from colon cells in the stool and is independent of the presence of blood in the stool, which is what FOBTs are based on. The potential of tests based on stool DNA lies in the detection of molecular changes occurring over a period of 7–10 years, which allows for earlier detection and better results [54,55]. (2) The other factor is imaging methods, namely MR colonography and CT capsules. The advantage of the first method over CT colonography is the lack of ionizing radiation. The second method uses a dissection-free X-ray imaging capsule which emits low-dose radiation beams using a rotating miniature electric motor. The results of the study by Gluck et al. for CT capsules indicate their potential practical application in the detection of CRC. A significant advantage of the tested method is its low invasiveness, low radiation (total exposure of patients was 0.03 ± 0.0007 mSv), and greater patient consent to the examination than in the case of a colonoscopy [50]. MR colonography has shown promising results in completed clinical trials as a tool helpful for detecting CRC at the level of 98.2%, with an overall sensitivity for detecting large polyps of 82% [56].

It should be noted that in order to prove that the pathologies detected by all of these methods are cancers, a colonoscopy should be performed with the collection of material for histopathological examination in order to identify the type of lesion detected. The ‘Computer Aided Detection of Polyps in Colonoscopy’ study (NCT04754347) checked the operation of the Skout device, which is a computer-aided tool for detecting polyps during colonoscopies in real time. The assumption of the study was the development of interval CRC as a result of missing polyps during a colonoscopy. The Skout tool was intended to improve the detection of adenomas in the study and thus reduce the number of cases of interval CRC. The system performs automated real-time analysis on endoscopic video data to identify potential polyps and produces an informational visual aid around the appropriate sections of the video frames on a display monitor [57]. Another system developed for polyp detection is the CADx system from the ‘Improving Optical Diagnosis of Colorectal Polyps Using CADx and BA-SIC’ study (NCT04349787), which aims to distinguish between benign and (pre-) malignant CRP by using state-of-the-art machine learning and archiving methods, namely deep learning textures [58].

## 4. Limitations

The included studies covered a five-year period (2019–2023) during the COVID-19 pandemic. Due to the epidemiological situation, the number of studies carried out during this period may differ from other periods. The need to focus on other research areas and other issues may have affected the current research shape. When looking at the results, readers should be aware that the duration of the research may have influenced its course and results. The second limitation is the lack of information on the financial effectiveness of individual tools. Financial efficiency was often not the subject of research. Additionally, the assessment is complicated by differences in the financing of health care systems around the world. Also, it is important to underline that as ClinicalTrial.Gov is the largest and one of the most-known databases, it does not include all of the research in the area of CRC screening and early detection.

## 5. Conclusions

The main trends in research on the early detection of colorectal cancer focus on improving tools to encourage participation in screening programs, as well as developing and enhancing tests for early detection. Universal screening remains one of the most effective tools for reducing the mortality and incidence of this disease. Current research is primarily directed at creating new methods based on circulating tumor DNA (ctDNA). However, the results from studies vary, indicating that the use of ctDNA in screening is still being explored. In particular, the high rate of false-positive results remains a significant challenge, warranting further investigation and solutions.

The second key area of research involves new techniques to improve the optical capabilities of endoscopic examinations, which aim to increase the accuracy of these tests. While these methods have shown promising results, their impact on screening costs and potential integration into nationwide screening programs still needs to be evaluated. Further simulations and calculations are required to assess their feasibility in large-scale settings.

It is also important to note that most research on colorectal cancer (CRC) is conducted in high-income countries, even though CRC is a global issue. In low-income countries, the disease burden is particularly high due to challenges in secondary prevention and delayed diagnosis.

Regarding strategies to increase participation in screenings, studies have consistently shown that social engagement strategies, such as multi-stage invitations in various audiovisual formats and tailored reminders, lead to higher patient participation. When combined with education on CRC risks, prevention, and testing, these solutions should be integrated as regular, active elements of the screening process.

Despite the availability of various screening tools and increasing health awareness, participation among individuals from at-risk groups remains low. Therefore, continued research into modern technologies is crucial to improving secondary prevention rates.

## Figures and Tables

**Table 1:** Clinical trials on CRC screening completed and ongoing in the period from 1 January 2019 to 31 December 2023.

Nr	Title ^a^	Status ^b^	Type ^c^	*N * ^d^	Intervention Used in Study ^e^	Category ^f^
1	Paired Promotion of Colorectal Cancer and Social Determinants of Health Screening (NCT04585919)	Completed	Interventional	26	Behavioral: Paired Screening Intervention	Population interventions aimed at increasing participation in screenings
2	Implementing Fit Kit Colorectal Cancer (CRC) Screening in High Risk Populations (NCT04848051)_	Completed	Interventional	3127	Behavioral: CRC Screening Reminder and Navigation Program	Population interventions aimed at increasing participation in screenings; educational interventions
3	Increasing Uptake of Bowel Screening (NCT05408169)	Completed	Interventional	40,000	Behavioral: Suggested Deadline for Return for Screening Test, Planning Sheet	Population interventions aimed at increasing participation in screenings; educational interventions
4	Flatbush FHC Proactive Colorectal Cancer Screening and Navigation (NCT05646355) [19]	Completed	Interventional	100	Behavioral: Proactive CRC Screening Outreach	Population interventions aimed at increasing participation in screenings
5	Screen to Save 2: Rural Cancer Screening Educational Intervention (NCT04414306) [21]	Completed	Interventional	382	Behavioral: Cancer Screening Education	Educational interventions
6	Colorectal Cancer Screening in Alaska Native Men NCT06436300)	Completed	Interventional	998	Behavioral: Motivational Text Messaging	Population interventions aimed at increasing participation in screenings
7	Lifestyle Intervention After Colonoscopy (LIFE-SCREEN Pilot) (NCT05273931)	Completed	Interventional	24	Behavioral: Lifestyle Intervention	Educational interventions
8	Veteran Primers for CRC Screening (NCT04923646)	Completed	Interventional	2404	Other: Primer	Population interventions aimed at increasing participation in screenings
9	Reminder Modality for CRC Screening (NCT05012007)	Completed	Interventional	2653	Behavioral: Reminder by Phone and Text	Population interventions aimed at increasing participation in screenings
10	Interventions to Improve Bowel Cancer Screening Uptake in Ireland: a 2 × 2 Factorial Trial (NCT05609396)	Completed	Interventional	8734	Behavioral: Behaviorally Enhanced Reminder Letter	Population interventions aimed at increasing participation in screenings
11	Using Behavioral Science to Improve Colorectal Cancer Screening Rates With Mailed FIT Kits (NCT04746469)	Completed	Interventional	2500	Behavioral: Delayed Automated Phone Call	Population interventions aimed at increasing participation in screenings
12	Study of Colorectal Cancer Screening Options (NCT05987709)	Completed	Interventional	400	Diagnostic Test: Guardant Shield Blood Test	Population interventions aimed at increasing participation in screenings
13	Effect of Patient Portal Messaging Before Mailing Fecal Immunochemical Testing Kit on Colorectal Cancer Screening Rates (NCT05115916)	Completed	Interventional	3880	Behavioral: Mychart Priming Message	Population interventions aimed at increasing participation in screenings
14	The Impact of Choice on Colorectal Cancer Screening (NCT05275530)	Completed	Interventional	20,509	Behavioral: Active Fit Choice, Active Colonoscopy Choice, and Active Dual Choice	Population interventions aimed at increasing participation in screenings
15	Multilevel Intervention Based on Colorectal Cancer (CRC) and Cervical Cancer Self-screening in Rural, Segregated Areas (NCT04471194) [22]	Completed	Interventional	48	Diagnostic Test: Self-Sampling Hpv Test, Fecal Occult Blood Test	Population interventions aimed at increasing participation in screenings; educational interventions
16	Text-based Colorectal Cancer Prevention Pilot (NCT05004376)	Completed	Interventional	118	Behavioral: Text Messaging	Population interventions aimed at increasing participation in screenings; educational interventions
17	Clinical Validation of An Optimized Multi-Target Stool DNA (Mt-sDNA 2.0) Test, for Colorectal Cancer Screening “BLUE-C” (NCT04144738)	Completed	Observational	26,758	Diagnostic Test: Mt-Sdna 2.0 Screening Test, Fit Test, Colonoscopy	Development, improvement, and application of early detection methods
18	Colorectal Cancer Screening Based on Predicted Risk (PRESENT) (NCT05357508) [23]	Completed	Interventional	515	Behavioral: Intervention Brochure	Population interventions aimed at increasing participation in screenings
19	Young-onset Colorectal Cancer Screening Based on Artificial Intelligence (NCT06342622)	Completed	Observational	11,000	Diagnostic Test: Using Routine Clinical Data and Machine Learning Models	Development, improvement, and application of early detection methods
20	Support Program for Adoption of Cancer Screening Interventions at a Rural Community-Based Organization (NCT04208724)	Completed	Interventional	35	Other: Training, Survey Administration	Population interventions aimed at increasing participation in screenings; educational interventions
21	Building Engagement Using Financial Incentives Trial—Colorectal Cancer Screening (NCT06124131)	Completed	Interventional	50	Other: Financial Incentive for Colorectal Cancer Screening	Population interventions aimed at increasing participation in screenings
22	Improving Cancer Screening and Follow-up in Community Health Centers (NCT05756725)	Completed	Interventional	8	Other: Practice-Level Implementation Strategies	Population interventions aimed at increasing participation in screenings
23	e-Motivacion: Developing and Pilot Testing an App to Improve Latinos’ Screening Colonoscopy Rates (NCT04987788)	Completed	Interventional	42	Behavioral: Video Education	Population interventions aimed at increasing participation in screenings; educational interventions
24	Incorporating ePrognosis for the Encouragement of Smarter Screening for Breast and Colorectal Cancer in Older Adults (CT05021172)	Completed	Interventional	75	Other: Best Practices, Survey Administration	Educational interventions
25	Combining Risk Factors and Faecal Immunochemical Testing in Colorectal Cancer Screening: a Randomized Controlled Trial (NCT04490551)	Completed	Interventional	6753	Diagnostic Test: Risk-Based Logistic Regression Model, Fit	Development, improvement, and application of early detection methods
26	CARES-HCV: Promoting Screening Uptake Among Diverse Baby Boomers (NCT04980157)	Completed	Observational	39	Behavioral: Cares-Hcv	Population interventions aimed at increasing participation in screenings; educational interventions
27	Blood and Stool Sample Collection in Subjects Participating in Colorectal Cancer Screening: Act Bold (NCT03821948)	Completed	Observational	5131	Diagnostic Test: Stool- and Blood-Based Biomarkers Associated with Genetic and Epigenetic Alterations	Development, improvement, and application of early detection methods
28	Plasma-based Colorectal Cancer Screening Research & Development (NCT04027790)	Completed	Observational	354	Diagnostic Test: Epi Procolon	Development, improvement, and application of early detection methods
29	Increasing Engagement With Messages Regarding CRC Screening Among Adults Aged 45–49 (NCT05281159)	Completed	Interventional	20,509	Behavioral: Risk Information	Population interventions aimed at increasing participation in screenings; educational interventions
30	Colonoscopy Screening: Setting Epic Follow-up Times (NCT05631873)	Completed	Interventional	43	Other: Assistance with Implementing Epic Smartlist	Educational interventions
31	Online Trial Examining Validity of the Shared Decision Making Process Survey with Video Vignettes (NCT04317274)	Completed	Interventional	401	Behavioral: Education with Materials	Educational interventions
32	Audio and Video Brochures for Increasing Colorectal Cancer Screening Among Adults Living in Appalachia (NCT05810714) [24]	Completed	Interventional	94	Other: Best Practices, Diagnostic Test (Fecal Immunochemical Test), Behavioral (Health Education)	Population interventions aimed at increasing participation in screenings
33	A Stool DNA Test for Detection of Advanced Colorectal Neoplasia in Asymptomatic Chinese Community Population (NCT04786704)	Completed	Observational	12,106	Diagnostic Test: Stool-Based Sdc2 Dna Methylation Test, Quantitative Fecal Immunochemical Test	Development, improvement, and application of early detection methods
34	Effectiveness of an Integrated Colorectal Cancer Screening in Saudi Arabia: A Pragmatic Randomized Trial (NCT04875793)	Completed	Interventional	300	Diagnostic Test: Stool for Occult Blood Test and Colonoscopy with Comprehensive Medical Checkups	Educational interventions
35	Health Service Intervention for the Improvement of Access and Adherence to Colorectal Cancer Screening (NCT04607291) [25]	Completed	Interventional	183	Other: Community Health Service	Population interventions aimed at increasing participation in screenings
36	Computer Aided Detection of Polyps in Colonoscopy (NCT04754347) [26]	Completed	Interventional	1472	Device: Computer-Aided Detection Device	Development, improvement, and application of early detection methods
37	Blood Collection Sub-Study of Exact Sciences Protocol 2019-01: “Clinical Validation of an Optimized Multi-Target Stool DNA (Mt-sDNA 2.0) Test, For Colorectal Cancer Screening BLUE-C” (NCT04144751)	Completed	Observational	23,494	Other: Blood Sample Collection	Educational interventions
38	Improving Colonoscopy Quality for Colorectal Cancer Screening in the National VA Healthcare System (NCT04389957)	Completed	Observational	500,000	Other: Quality of Screening for Veterans	Development, improvement, and application of early detection methods
39	Exploring the Risk Factors for Colorectal Cancer in Our General Population Using Asian Pacific Screening Score: a Survey From Pakistan (NCT05617378)	Completed	Observational	558	Other: Asian Pacific Screening Score	Educational interventions
40	Evaluation of Letters Promoting Colorectal Cancer Testing (NCT04548765)	Completed	Interventional	14,644	Behavioral: Letter, Loss Frame, and Fear Appeals, Enhanced Fear Appeals, and Decoy Effect	Population interventions aimed at increasing participation in screenings
41	CB-17-08 Augmented Endoscopy System for Mucosal Lesion Detection During Colonoscopy for Colon Rectal Cancer (NCT03954548)	Completed	Interventional	249	Device: Cb-17-08 Cade	Development, improvement, and application of early detection methods
42	Optimising Colorectal Cancer Screening Participation (NCT04292366) [27]	Completed	Interventional	60,000	Behavioral: Invitation Procedure	Population interventions aimed at increasing participation in screenings
43	Usefulness of GI-GENIUS in FIT-based Colorectal Cancer Screening Program (NCT04673136)	Completed	Interventional	3400	Device: Gi-Genius Medtronic|Other: Colonoscopy	Development, improvement, and application of early detection methods
44	Endocuff Enhanced Colonoscopy: Does it Improve Polyp Detection and Make Rectal Retroflexion Unnecessary (NCT05615857)	Completed	Interventional	750	Device: Use of Endocuff During Colonoscopy	Development, improvement, and application of early detection methods
45	Identification of New Diagnostic Protein Markers for Colorectal Cancer (EXOSCOL01) (NCT04394572) [28]	Completed	Observational	80	Diagnostic Test: Protein Markers	Development, improvement, and application of early detection methods
46	A Video Intervention to Decrease Patient Fear of Colonoscopy After a Positive Fecal Immunochemical Test (NCT05458986) [29]	Completed	Interventional	66	Other: Media Intervention	Educational interventions
47	Motility of the Check-cap’s MD1 Colon Capsule in Subjects Following Colon Rectal Cancer Screening by Colonoscopy (NCT03785665)	Completed	Interventional	88	Device: MDI	Development, improvement, and application of early detection methods
48	Effect of Adding Simethicone to Split-dose Polyethylene Glycol for Bowel Preparation in a Screening Colonoscopy Setting (NCT03816774)	Completed	Interventional	412	Drug: Peg Split-Dose and Simethicone	Development, improvement, and application of early detection methods
49	An Interactive Video Educational Tool Improves the Quality of Bowel Preparation for Colonoscopy (NCT04491565) [30]	Completed	Interventional	270	Behavioral: Educational Video	Educational interventions
50	Lactulose vs. Polyethylene Glycol as Bowel Preparation for Colonoscopy in Adults (NCT05726344)	Completed	Interventional	150	Drug: Lactulose, Polyethylene Glycol 3350	Development, improvement, and application of early detection methods
51	Text Message-Based Nudges Prior to Primary Care Visits to Increase Care Gap Closure (NCT05799976)	Completed	Interventional	29,334	Behavioral: Text Message Nudge	Population interventions aimed at increasing participation in screenings
52	The diagnostic Value of Serum autotaxin Level And Colorectal Cancer (NCT06091592)	Completed	Observational	129	Other: Serum Autotaxin Levels	Development, improvement, and application of early detection methods
53	Feasibility Study of a Novel Single Use Robotic Colonoscopy System (NCT03979690)	Completed	Interventional	20	Device: Nisinspire-C System	Development, improvement, and application of early detection methods
54	Evaluation C-Scan System in Providing Structural Information and Polypoid Lesions in the Colon of Healthy Subjects (NCT04038736)	Completed	Interventional	82	Device: C-Scan System	Development, improvement, and application of early detection methods
55	The Effect of Bowel Preparation Training Given to Patients Undergoing Colonoscopy (training) (NCT06159855) [31]	Completed	Interventional	80	Behavioral: Nurse-Led Education For Bowel Preparation for Colonoscopy	Educational interventions
56	Pharmacogenomics Testing in Directing the Optimal Use of Supportive Care Medications in Patients With Stage III–IV Cancer NCT04067960)	Completed	Interventional	197	Procedure: Biospecimen Collection, Genetic Testing; Other: Quality-of-Life Assessment	Development, improvement, and application of early detection methods
57	A Multicenter Clinical Trial of Stool-based DNA Testing for Early Detection of Colon Cancer in China (NCT04722055) [32]	Completed	Observational	1273	Diagnostic Test: Multigene Methylation Detection Kit	Development, improvement, and application of early detection methods
58	Ellagic Acid, Urolithin and Colonic Microbial Communities Affected by Walnut Consumption (NCT04066816)	Completed	Interventional	47	Other: Food and Nutricion	Educational interventions
59	Improving Optical Diagnosis of Colorectal Polyps Using CADx and BASIC (NCT04349787) [33]	Completed	Observational	60	Device: Computer-Aided Diagnosis (CADx)	Development, improvement, and application of early detection methods
60	Test Up Now Education Program (TUNE-UP) (NCT04304001) [34]	Completed	Interventional	115	Behavioral: Community Health Advisor	Population interventions aimed at increasing participation in screenings; educational intervntions
61	Implementing a Multilevel Intervention to Accelerate Colorectal Cancer Screening and Follow-up (NCT04514341)	Ongoing	Observational	74,560	Other: Multilevel Intervention to Increase Rates of CRC Screenings, Follow-Ups, and Referrals to Care in Federally Qualified Health Centers (FQHCs)	Population interventions aimed at increasing participation in screenings; educational interventions
62	Educate, Assess Risk and Overcoming Barriers to Colorectal Screening Among African Americans (NCT03640208) [35]	Ongoing	Interventional	150	Behavioral: Education Presented by Trained Caregiver	Population interventions aimed at increasing participation in screenings; educational interventions
63	Colorectal Cancer Screening Assessment Study (NCT04500171)	Ongoing	Interventional	262	Behavioral: Colorectal Cancer Screening for Life Brochure; Sam + Cam Smog, Colocare Instruction Video; Modified Sam + Cam, Superior Rating	Educational interventions
64	Engaging Black Men in Colorectal Cancer Screening (NCT05980182)	Ongoing	Interventional	30	Device or Behavioral: mHealth Application	Population interventions aimed at increasing participation in screenings
65	Scaling CRC Screening Through Outreach, Referral, and Engagement (SCORE) (NCT04406714)	Ongoing	Interventional	4318	Behavioral: Trial Mailed Fit Intervention	Population interventions aimed at increasing participation in screenings
66	Screening More Patients for Colorectal Cancer Through Adapting and Refining Targeted Evidence-Based Interventions in Rural Settings, SMARTER CRC (SMARTER CRC) (NCT04890054) [36]	Ongoing	Interventional	15,510	Behavioral: Patient Navigation, Diagnostic Test: Fecal Immunochemical Test	Population interventions aimed at increasing participation in screenings
67	Colorectal Cancer Screening Intervention Study (NCT06424197)	Ongoing	Interventional	799	Behavioral: Racial Group-Targeted Messages	Educational interventions
68	Direct Information to At-risk Relatives (DIRECT) (NCT04197856) [37]	Ongoing	Interventional	490	Other: Standard Care Encouraging Family-Mediated Disclosure of Hereditary Cancer Risk	Educational interventions
69	Community Collaboration to Advance Racial/Ethnic Equity in CRC Screening (NCT05714644)	Ongoing	Interventional	5255	Diagnostic Test: Fit Kit Screening Test, Cologuard Screening Test	Population interventions aimed at increasing participation in screenings
70	Accelerating Colorectal Cancer Screening Through Implementation Science in Appalachia (NCT04427527)	Ongoing	Interventional	5425	Behavioral: Multi-Level Intervention to Increase CRC Screenings	Population interventions aimed at increasing participation in screenings
71	Understanding Patient Preference on Colorectal Cancer Screening Options (NCT05536713)	Ongoing	Observational	500	Diagnostic Test: Guardant Shield Blood-Based Colorectal Cancer Screening Test	Development, improvement, and application of early detection methods
72	General Practitioners and Participation Rate in ColoRectal Cancer Screening (AMDepCCR) (NCT04492215) [38]	Ongoing	Interventional	902	Behavioral: Interaction between Patient and Trained General Practitioners in Motivational Interviewing	Population interventions aimed at increasing participation in screenings
73	Stool DNA to Improve Colorectal Cancer Screening Among Alaska Native People (NCT04336397)	Ongoing	Interventional	1447	Diagnostic Test: Multi-Target Stool DNA Test	Development, improvement, and application of early detection methods
74	Connecting Black Families in Houston, Texas to Hereditary Cancer Genetic Counseling, Genetic Testing, and Cascade Testing by Using a Simple Genetic Risk Screening Tool and Telegenetics (NCT05694559)	Ongoing	Interventional	1000	Other: Genetic Testing and Counseling	Development, improvement, and application of early detection methods
75	A Smartphone-Based Intervention to Improve Colorectal Cancer Screening in African American Men (NCT06052202)	Ongoing	Interventional	158	Device or Behavioral: CRC Mhealth	Population interventions aimed at increasing participation in screenings; educational interventions
76	The PRIME-CRC Trial to Promote CRC Screening in Rural Communities (NCT04313114) [20]	Ongoing	Interventional	800	Behavioral: Health Literacy Appropriate Education and Demonstration	Population interventions aimed at increasing participation in screenings; educational interventions
77	Informed Choice—Compass (NCT05246839)	Ongoing	Observational	5280	Behavioral: Brief Video Education	Population interventions aimed at increasing participation in screening
78	Shared Decision-making and Colorectal Cancer Screening (NCT04748380)	Ongoing	Interventional	60	Other: CRC Decision Aid Pamphlet, Home Safety Pamphlet	Population interventions aimed at increasing participation in screenings; educational interventions
79	Aim 3, Optimizing CRC Screening in Patients with Diabetes in Safety-net Primary Care Settings (NCT05785780)	Ongoing	Interventional	30	Behavioral: Targeted CRC Screening Toolkit	Population interventions aimed at increasing participation in screening; educational interventions
80	Lifestyle Intervention Among Participants of the French Colorectal Cancer Screening Program (NCT04257526)	Ongoing	Interventional	500	Behavioral: Diet and Lifestyle Advice Following a Positive Fit Test and Diagnostic Colonoscopy	Educational interventions
81	Validation of Advanced Colorectal Neoplasm Risk Categories in a Prospective Cohort in Mexico (NCT05661292)	Ongoing	Observational	2000	Diagnostic Test: Fecal Inmunochemical Test, Colonoscopy	Educational interventions
82	Blood-Based Colorectal Cancer (CRC) Screening Implementation into Clinical Practice Highlands (NCT06119425)	Ongoing	Interventional	600	Diagnostic Test: Blood-Based CRC Screening	Population interventions aimed at increasing participation in screenings
83	A Prospective Cohort Study on Colorectal Cancer Screening in Community Population (NCT05485077)	Ongoing	Observational	18,000	Diagnostic Test: Polygene Methylation Detection Technology	Development, improvement, and application of early detection methods
84	The Purpose of This Study is to Determine the Frequency of Colorectal Cancer in Male and Female Endurance Athletes Between the Ages of 35 and 50 (NCT05419531)	Ongoing	Observational	100	Other: Screening and Follow-up Colonoscopy	Educational interventions
85	Evaluation of the ctDNA LUNAR Test in an Average Patient Screening Episode (NCT04136002)	Ongoing	Observational	44,467	Diagnostic Test: Blood-Based ctDNA LUNAR-2 Test	Development, improvement, and application of early detection methods
86	Colorectal Cancer Awareness, Research and Education and Screening—Rural Expansion, Access and Capacity for Health (NCT04464668)	Ongoing	Observational	49	Other: Provider Tools, Provider Surveys	Population interventions aimed at increasing participation in screenings; educational interventions
87	Comparing the Effectiveness of Written vs. Verbal Advice for First Degree Relatives of Colorectal Cancer Patients (NCT06242197) [39]	Ongoing	Interventional	180	Behavioral: Written Advice	Educational interventions
88	Test Up Now Education Program (NCT04304001)	Ongoing	Interventional	115	Behavioral: Community Health Advisor	Educational interventions
89	Implementation Research to Increase Colorectal Cancer Screening Rates Among Low Income and Ethnic Minority Groups (NCT06090643)	Ongoing	Interventional	4000	Other: Best Practices; Behavioral: Educational Intervention; Device: Electronic Health Record Review; Diagnostic Test: Fecal Immunochemical Test	Population interventions aimed at increasing participation in screenings; educational interventions
90	Polyprev: Study to Compare Fecal Immunochemical Test With Endoscopic Surveillance After Advanced Adenoma Resection in Fecal Immunochemical Test Colorectal Cancer Screening Programs. (CT04967183)	Ongoing	Interventional	3788	Diagnostic Test: Annual Fit	Development, improvement, and application of early detection methods
91	A Community Population Screening Cohort Study Based on Polygene Methylation Detection for Colorectal Cancer in Yangzhou (NCT05336539)	Ongoing	Observational	80,000	Diagnostic Test: Polygene Methylation Detection Technology	Development, improvement, and application of early detection methods
92	Community Health Workers United to Reduce Colorectal Cancer and Cardiovascular Disease Among People at Higher Risk (NCT05174286)	Ongoing	Interventional	880	Behavioral: Education; Other: System Performance	Population interventions aimed at increasing participation in screenings; Educational interventions
93	Blood Test (Guardant Shielda) for Screening of Colorectal Cancer in Underserved Patients (NCT05716477)	Ongoing	Observational	300	Procedure: Biospecimen Collection	Development, improvement, and application of early detection methods
94	Effectiveness of Modified Integrated Colorectal Cancer Screening System in Saudi Arabia (NCT05785975)	Ongoing	Interventional	2520	Other: System Performance	Population interventions aimed at increasing participation in screenings
95	A Multi Center Study Comparing the Efficacy of CAD EYE and the Standard of Care (White Light) (NCT05523271)	Ongoing	Interventional	1000	Diagnostic Test: Cad Eye (Computer-Aided Diagnosis)	Development, improvement, and application of early detection methods
96	Eliminating Barriers to Colorectal Cancer Screening Using Rapid Cycle Testing: A Pilot Study (CT05524428)	Ongoing	Interventional	2	Other: System Strategy	Population interventions aimed at increasing participation in screenings
97	Addressing Disparities in Colorectal Cancer Screening in Black and Underserved Phoenix Communities (NCT05447923)	Ongoing	Interventional	450	Behavioral: Educational Intervention; Diagnostic Test: Fecal Immunochemical Test	Population interventions aimed at increasing participation in screenings; educational interventions
98	Virtual Colonoscopy Using Omnipaque as a Contrast Agent (CT04582500)	Ongoing	Observational	50	Drug: Iohexol	Development, improvement, and application of early detection methods
99	A Feasibility Study to Improve Colorectal Cancer Screening Among Racially Diverse Zip Codes in a Persistent Poverty County Using Navigation and Machine Learning Predictive Algorithms (NCT05383976)	Ongoing	Observational	200	Other: Machine Learning Algorithm with Existing Penn Medicine CRC Patient Navigation Program	Population interventions aimed at increasing participation in screenings
100	A Community-Based Educational Intervention to Improve Colorectal Cancer Screening (NCT04392050)	Ongoing	Observational	90	Behavioral: Education	Population interventions aimed at increasing participation in screenings; educational interventions
101	A Study of Using Social Networks to Encourage Three Peers to Complete Screening for Colorectal Cancer (NCT05793593)	Ongoing	Interventional	80	Other: Fitx3 Intervention and CRC Education	Population interventions aimed at increasing participation in screenings
102	Early Screening of Colorectal Cancer Based on Plasma Multi-omics Combining With Artificial Intelligence (NCT05587452)	Ongoing	Observational	950	Diagnostic Test: Colonoscopy, Test Of ctDNA Methylation	Development, improvement, and application of early detection methods
103	Clinical Efficacy Evaluation of a Computer-aided Colonoscopy as Compared With the Standard Colonoscopy (NCT05240625)	Ongoing	Interventional	1500	Device: “Aetherai” Computer-Aided Polyp Detection (Cade) Systems For Colonoscopy	Development, improvement, and application of early detection methods
104	Colonoscopy Outreach for Rural Communities Aim 2 (NCT05453630)	Ongoing	Interventional	527	Behavioral: Patient Navigation	Population interventions aimed at increasing participation in screenings; educational interventions
105	A Cross-sectional Partnership to Improve Prevention (NCT05903885)	Ongoing	Interventional	1200	Other: On-Site Fecal Immunochemical Test (Fit) Kit Distribution	Population interventions aimed at increasing participation in screenings; educational interventions
106	Online Mindfulness-Based Intervention to Decrease Pre-Procedural Anxiety Before a First-Time Screening Colonoscopy (NCT06233253)	Ongoing	Interventional	100	Other: Best Practices, Medical Chart Review; Behavioral: Online Mindfulness Meditation	Educational interventions
107	Prevention of Colorectal Cancer Through Multiomics Blood Testing (PREEMPT CRC) (NCT04369053) [40]	Ongoing	Observational	50,000	Diagnostic Test: Freenome Test	Development, improvement, and application of early detection methods
108	Evaluation of a Model for the Early Diagnosis of Colorectal Cancer by the Detection of 5-hydroxymethylcytosine (5-hmC) in Plasma Cell-free DNA to the Community Colorectal Cancer Screening Program (NCT05638243)	Ongoing	Observational	10,000	Diagnostic Test: 5-hydroxymethylcytosine (5-hmC) in Plasma Cell-Free DNA	Development, improvement, and application of early detection methods
109	Impact of Colorectal Cancer and Nutrition Education Program Among Minority Patients With Type 2 Diabetes (NCT05765214) [41]	Ongoing	Interventional	120	Behavioral: Education	Educational interventions
110	Diagnostic Accuracy of a Panel of Bacterial Gene Markers (M3) for Colorectal Advanced Neoplasia (NCT05405673)	Ongoing	Observational	2500	Diagnostic Test: Fecal Immunochemical Test (FIT)	Development, improvement, and application of early detection methods
111	Educate, Assess Risk and Overcoming Barriers to Colorectal Screening Among African Americans (NCT03640208)	Ongoing	Interventional	150	Behavioral: Education Presented by Trained Caregiver	Population interventions aimed at increasing participation in screenings; educational interventions
112	Prospective Screening and Differentiating Common Cancers Using Peripheral Blood Cell-Free DNA Sequencing (NCT06036563)	Ongoing	Observational	3200	Diagnostic Test: Peripheral Blood Cell-Free DNA Sequencing	Development, improvement, and application of early detection methods
113	Colorectal Cancer and Pre-Cancerous Adenoma Non-Invasive Detection Test Study (NCT04739722)	Ongoing	Interventional	8000	Diagnostic Test: Colosense Stool Sample Collection Kit	Development, improvement, and application of early detection methods
114	Early Detection of Advanced Adenomas and Colorectal Cancer (NCT06342440)	Ongoing	Observational	2000	Diagnostic Test: DENEB	Development, improvement, and application of early detection methods
115	Mucosa Adherent Intestinal Microbiome in Microscopic Colitis and Colorectal Cancer (NCT06172647)	Ongoing	Observational	60	Procedure: Colonoscopy	Development, improvement, and application of early detection methods
116	Detection of Cancer in Breath Samples by Trained Detection Dogs (CT06255041)	Ongoing	Observational	1250	Other: Training Dogs in Breath Detection	Development, improvement, and application of early detection methods
117	Collection of Samples USOPTIVAL Study (NCT04792684)	Ongoing	Observational	1300	Diagnostic Test: Optimization of Plasma Circulating Free DNA (cfDNA) Marker Panel	Development, improvement, and application of early detection methods
118	Exogenous and Endogenous Risk Factors for Early-onset Colorectal Cancer (NCT05732623)	Ongoing	Observational	2300	Behavioral: Semi-Quantitative Food Frequency Questionnaire (SQFFQ)	Educational interventions
119	Project CADENCE (CAncer Detected Early caN be CurEd) (NCT05633342)	Ongoing	Observational	15,000	Other: microRNA Expression in Tandem with Other Biomarkers	Development, improvement, and application of early detection methods
120	cfDNA Assay Prospective Observational Validation for Early Cancer Detection and Minimal Residual Disease (NCT05366881)	Ongoing	Observational	7000	Diagnostic Test: Blood-Based, Multi-Cancer Screening Tests	Development, improvement, and application of early detection methods
121	Collecting Blood Samples From Patients with and Without Cancer to Evaluate Tests for Early Cancer Detection (NCT05334069)	Ongoing	Observational	2000	Other: Questionnaire Administration; Procedure: Biospecimen Collection	Development, improvement, and application of early detection methods

^a^ The study title. ^b^ Status of the study—ongoing or completed—based on the study’s completion date (date on which the last participant in a clinical study was examined or received an intervention or treatment to collect final data for the primary outcome measures, secondary outcome measures, and adverse events (that is, the last participant’s last visit)). ^c^ Describes the nature of a clinical study. Study types include interventional studies (also called clinical trials), observational studies (including patient registries), and expanded access. ^d^ The number of participants in a clinical study. The “estimated” enrollment is the target number of participants which the researchers needed for each study. ^e^ A process or action which is the focus of a clinical study. Interventions include drugs, medical devices, procedures, vaccines, and other products which are either investigational or already available. Interventions can also include noninvasive approaches, such as education, modifying one’s diet, and exercise. ^f^ Intervention category defined in the study: (1) population interventions aimed at increasing participation in screenings; (2) educational intervention; and (3) development, improvement, and application of early detection methods.

## Data Availability

Data available on request from authors.
